# Study on Self-Leveling of Foamed Concrete for Long-Distance-Tunnel-Gas-Pipeline Backfill

**DOI:** 10.3390/polym14142886

**Published:** 2022-07-16

**Authors:** Chunbao Li, Xiaotian Li, Shen Li, Di Guan, Chang Xiao, Yun Lei, Valentina Y. Soloveva, Hojiboev Dalerjon, Pengju Qin, Xiaohui Liu

**Affiliations:** 1 College of Pipeline and Civil Engineering, China University of Petroleum (East China), Qingdao 266580, China; z21060136@s.upc.edu.cn (X.L.); z21060135@s.upc.edu.cn (C.X.); 2 Construction Project Management Branch, National Petroleum and Natural Gas Pipeline Network Group Co., Ltd., Langfang 065000, China; lishen@pipechina.com.cn (S.L.); guandi@pipechina.com.cn (D.G.); 3 Henan Huatai New Material Technology Corp., Ltd., Nanyang 473000, China; jennifer@gmail.com; 4 Emperor Alexander IST Petersburg State Transport University, 190031 St. Petersburg, Russia; soloviova-pgups@mail.ru; 5 Mining-Metallurgical Institute of Tajikistan, Buston City 735730, Tajikistan; dalerkhojiboev@gmail.com; 6 School of Civil Engineering, Taiyuan University of technology, Taiyuan 030024, China; qinpengju2017@126.com; 7 Qingdao Urban Development Group Co., Ltd., Qingdao 266011, China; liuxiaohui2014upc@163.com

**Keywords:** foamed concrete, concrete-mix proportion, orthogonal test, self-leveling-construction technology

## Abstract

In the foamed-concrete-backfilled-gas-pipeline project, the fluidity of foamed concrete has a great impact on the construction quality. This research studied the fluidity of foamed concrete through laboratory tests. By changing the water–cement ratio, admixtures, additives, foaming-agent content and other test parameters, foamed concrete with different fluidities was prepared, and the effects of the above parameters on the fluidity of foamed concrete were analyzed. At the same time, the construction equipment was improved in the three steps of transportation, production and pouring. The results show the factors affecting the fluidity of foamed concrete are, in order of importance, foaming-agent content > water–cement ratio > water-reducer content > admixture content. According to the orthogonal-test results, the control scheme meeting the fluidity requirements of the actual engineering project had gains as follows: the water–cement-ratio range from 0.5 to 0.6, the amount of admixture from 35% to 40%, the water-reducer content at 1% and the foaming-agent content from 3% to 3.5% so as to ensure the automatic leveling of foamed concrete under the best flow state.

## 1. Introduction

**Figure 1:**
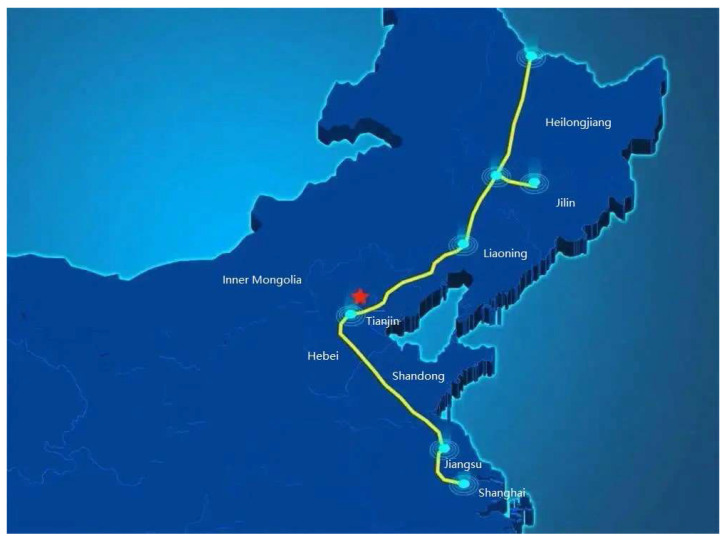
The overall route map of the China–Russia East-Route pipeline.

The China–Russia East Route is a long-distance natural-gas pipeline with the largest diameter and the highest pressure in China and even the world. Its trunk route starts at Heihe City, Heilongjiang Province; passes through nine provinces, including Heilongjiang, Jilin, Inner Mongolia, Liaoning, Hebei, Tianjin, Shandong, Jiangsu and Shanghai and ends at Baihe Town, Shanghai. The total length of the trunk route is 3334.6 km, the designed transmission capacity 380 × 108 m^3^/a, the designed pressure 12 MPa/10 MPa and the pipe diameter 1422 mm/1219 mm/1016 mm. The overall route is shown in Figure 1. Crossing the Yangtze River, the China–Russia East-Route natural-gas pipeline is located in Haimen Economic and Technological Development Zone, Nantong City, Jiangsu Province and Changshu Economic and Technological Development Zone, Jiangsu Province. It adopts the shield-tunnel-crossing scheme. This intercity project is of large scale. The horizontal length of the line within the crossing design range is 10,324 m, both sides of which are crossing sections. The crossing horizontal length of the shield tunnel is 10,226 m, which is the control project of the China–Russia East Route.

**Figure 2:**
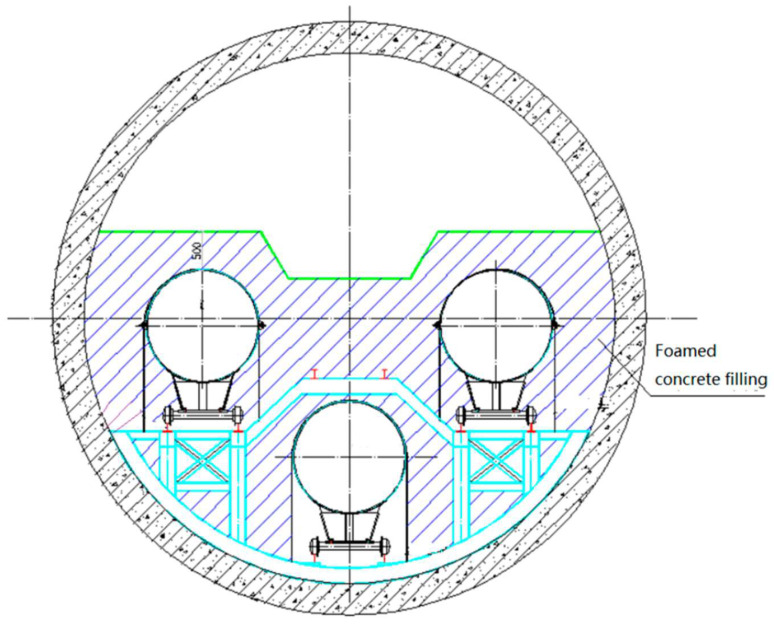
Schematic diagram of foamed-concrete tunnel filling.

According to the design scheme of the project, three D1422 mm pipelines are laid in the Yangtze River Shield Tunnel, one for the China–Russia East-Route pipeline, one for the Jiangsu coastal pipeline and one for the reserved pipeline shared by both parties. According to the requirements of the Engineering Construction Project Department of the National Pipe Network Group, the tunnel shall be backfilled after the pipeline installation in the tunnel is completed, as shown in Figure 2.

The common backfilling methods for gas-pipeline tunnels include water backfilling, air backfilling and foamed-concrete backfilling. Water backfilling is widely used due to its convenient construction and maintenance, but it has a great impact on the tunnel structure. For air backfilling, the tunnel section must be extended to ensure that there is enough inspection space inside. In addition, air backfilling also calls for channels, ventilation facilities, lighting systems, gas detection, etc., and mandatory measures to prevent pipeline expansion or contraction caused by temperature changes, which will increase the project investment. Therefore, the foamed-concrete-self-leveling-backfilling method is adopted in this project.

Foamed concrete has advantages such as light weight and thermal insulation. With this method, the pipeline can be prevented from being damaged by external forces and daily maintenance. The pipeline has good restraint and structural seismic performance. It can not only improve the safety of pipeline operation but also facilitate pipeline operation and management. Foamed concrete is a lightweight porous concrete made of cement, the main cementitious material. After introducing bubbles into the slurry made of aggregates, additives, water and other components, it is mixed, poured and cured. At present, it is widely used in the fields of pipeline backfilling, roof insulation, cushioning and energy absorption [1,2,3,4,5]. Song Qiang et al. [6] made a detailed review on the research progress and application of foamed concrete.

Scholars at home and abroad have done a lot of research on foamed concrete. Xin Yucong et al. [7] have studied the engineering performance of fiber-reinforced foamed concrete, and thus obtained the influence of fiber reinforcement on the fluidity of foamed concrete. Zhu Junjie et al. [8] used ordinary portland cement as cementitious material, mixed with polycarboxylate superplasticizer, and prepared foamed concrete by physical foaming technology. They studied the influence of the content of polycarboxylate superplasticizer on the compressive strength, fluidity and other properties of foamed concrete. Relying on practical projects, Yang Weihui [9] studied the application of foamed concrete in cavity filling, which provided a lot of theoretical basis for this study. On the basis of traditional foamed concrete, Shang Xiaoyu et al. [10] developed a new type of foamed concrete by adding new admixtures, reflecting on the importance of sustainable and multi-functional development of lightweight concrete for buildings under the background of global climate change. Lu Jianglong et al. [11,12,13,14,15,16,17,18] redesigned and adjusted the mix proportion of foamed concrete to improve its engineering properties, including fluidity.

Taking the research results of scholars at home and abroad into consideration, it is found that the self-leveling-construction method of foamed concrete has high requirements for fluidity and on-site pouring equipment. To realize the automatic leveling of foamed concrete in construction, it is necessary to optimize the mix proportion of foamed concrete.

Niu Yunhui [19] found in the research of self-leveling foamed concrete that the traditional foamed concrete paste has low density, high viscosity and poor fluidity. During the construction, when the foamed concrete pouring position reached the designed height, a long scraper was used for manual leveling, which increased the construction risk and caused artificial disturbance.

Therefore, this paper first carried out the test of foamed-concrete-fluidity determination, and determined the mix proportion range when the fluidity of foamed concrete reached the maximum. Then, through the investigation of the project and referring to the existing construction technology and methods, the working mode of the construction equipment was optimized, and a new foamed concrete production and backfilling system was designed to ensure that the bulk cement can be transported to the production vehicle in time and in sufficient quantity so as to produce the foamed concrete immediately and use it to cast in place without delay.

The new foamed-concrete-production-and-backfilling system was combined with foamed concrete with high fluidity to play a great role in the backfilling construction of natural-gas pipelines in long-distance and confined-space tunnels. Thus the poured foamed concrete can level automatically and construction risks and human disturbance can be reduced so as to make the whole pouring process almost automated.

## 2. Experimental Procedure

### 2.1. Raw Materials

Cement is an important cementitious material of concrete, and the hydration of cement is the basis for the hardening of concrete. Therefore, the performance of cement has a great impact on the performance of concrete. The cement used was ordinary portland cement from Huaxin Cement (Nantong) Co., LTD., Huaxin Cement Co., Ltd., located in Nantong, China, whose physical indicators are shown in Table 1 and comply with GB 175 [20].

Adding a certain amount of HTFC foamed-concrete admixture into ordinary portland cement can improve the problems of easy pulverization and cracking of foam concrete in the later stage and reduce the hydration of the system at the same time. HTFC foamed-concrete admixtures (hereinafter referred to as “HTFC admixtures”) were selected as admixtures. HTFC admixtures are nano-level inorganic mineral fine powder formed by compounding and evenly mixing with a variety of minerals as the main raw materials. The HTFC manufacturer is Henan Huatai New Material Technology Co., Ltd., which is located in Henan, China.The testing standard is *GB/T51003-2014 Technical code for Application of Mineral Admixtures* [21]. HTFC admixtures contain fly ash, the chemical composition of which is shown in Table 2. 

The water-reducer agent can significantly improve the fluidity of foamed concrete with the same mix proportion, significantly reduce the amount of water used with the same fluidity and cement dosage, improve the strength and durability of concrete and effectively reduce the cost. Polycarboxylate superplasticizer was selected as the water-reducer agent, The manufacturer of water reducing agent is Laiyang Hongxiang construction admixture factory, which is located in Laiyang City, China, and its indexes are shown in Table 3. It complies with GB 8067.

**Figure 3:**
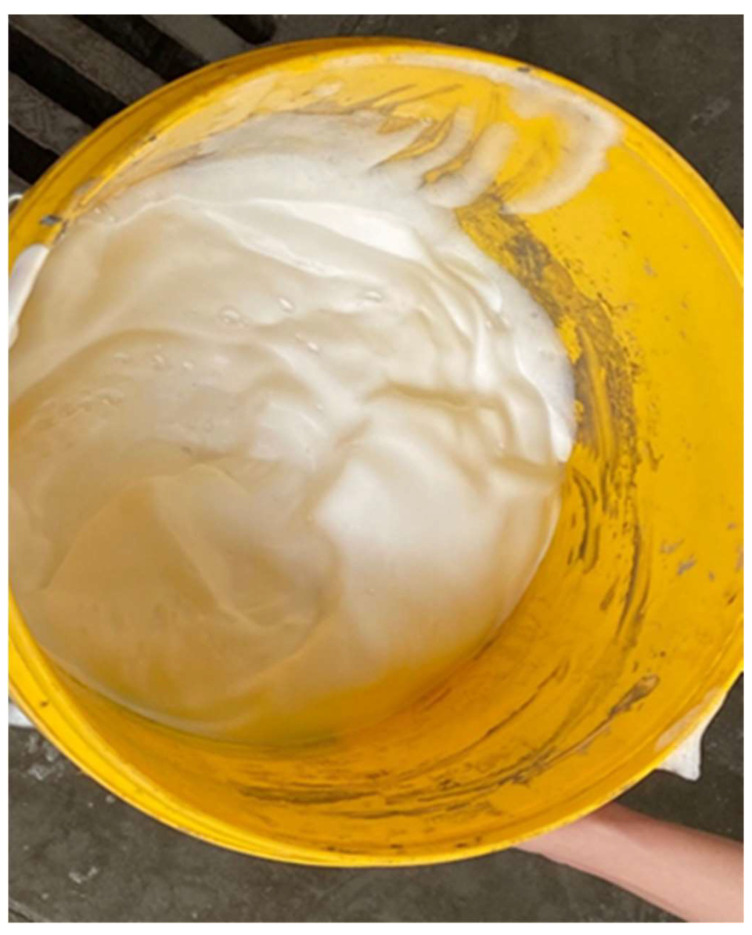
The foam produced by physical foaming with this foaming agent.

Foaming agent is one of the main additives in the production and preparation of foamed concrete, which has an important impact on the physical and mechanical properties and pouring constructability of foam concrete. In this experiment, HT foamed-concrete-foaming agent was adopted. The manufacturer of foaming agent is Henan Huatai New Material Technology Co., Ltd., which is located in Henan, China. Figure 3 shows the foam produced by physical foaming with this foaming agent.

The water used for mixing foamed concrete was tap water from Qingdao City, which conforms to the requirements in *Standard of Water for Concrete (JGJ 63-2006)* [22].

### 2.2. Test Equipment

**Figure 4:**
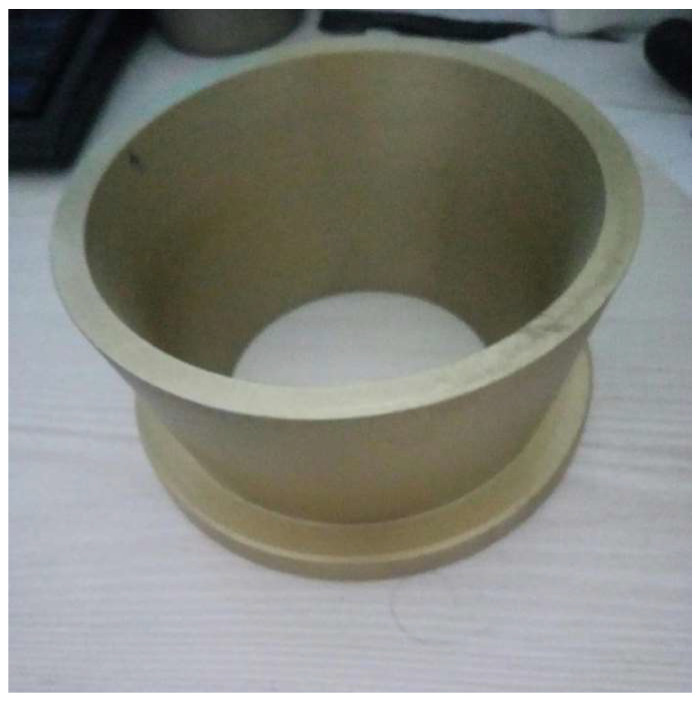
Truncated-cone round die.

Foaming machine: Bl168-8, Hefei Baile Energy Equipment Co., Ltd., Hefei, China; Mixer: HJW60, Wuxi Jianyi Instrument Machinery Co., Ltd., Wuxi, China; Truncated-cone round die: 100 × 70 × 60 large clean slurry testing die with upper and lower caliber and height of 70 mm, 100 mm and 60 mm, respectively. Thetruncated-cone round die is shown in Figure 4.

### 2.3. Orthogonal Test Design

The orthogonal test [23,24,25] fully considered the interacted influence of HTFC admixtures, foaming agent, water–cement ratio, water-reducer agent and other factors on the fluidity of foamed concrete and analyzed the respective effect of different factors on the fluidity of foamed concrete. Water–cement ratio was defined as factor A, HTFC admixture was defined as factor B, water-reducer agent was defined as factor C and foaming agent was defined as factor D. The factor level of orthogonal test is shown in Table 4 below. 

### 2.4. Preparation of Foamed Concrete

First, HTFC admixtures and other cementitious materials, as well as other materials except foaming agent, were evenly mixed, and the water-reducer agent was dissolved completely in water, which then was added to the uniformly stirred cementitious material and stirred for about 3 min. HT composite foaming agent was added while maintaining high-speed stirring for about 60 s.

### 2.5. Fluidity Determination Method

The smooth-glass plate, truncated-cone round die, flat cutter and measuring cup were cleaned and wiped, and then the truncated-cone die was placed on the smooth-glass plate, while the sample measured by measuring cup was poured slowly in the truncated-cone round die. The truncated-cone round die was tapped by fingers outside to fill it completely. The flat cutter was used to scrape along the port plane of the round die. Then slowly the truncated-cone round die was lifted vertically with both hands and kept there for 1 min. Finally, the maximum horizontal diameter was measured with a vernier caliper; thus the fluidity was obtained.

### 2.6. Orthogonal-Test Scheme and Results

There are four factors, including water–cement ratio, HTFC admixtures dosage, water-reducer-agent content and foaming-agent content, and three levels were set for each factor. Therefore, the orthogonal test of L9(3^4^) with four factors and three levels was designed, as shown in Table 5. The results of orthogonal-test data are shown in Table 6.

## 3. Test Results and Analysis

### 3.1. Range Analysis of Orthogonal Test

Range analysis was conducted on the orthogonal-test results [26], and the range analysis table of the orthogonal test is shown in Table 7.

The range value reflects the degree of influence of changes in the level of each factor on the test results. The greater the range is, the greater the influence on the assessment index will be when the level of the factors in the column changes, which is called the most important factor. According to the data in Table 7, among the factors that have an important influence on the fluidity of foamed concrete, the order of importance is D > A > C > B. That is, dosage of foaming agent > water–cement ratio > dosage of water-reducer agent > dosage of HTFC admixture. It can be found that foaming agent has the most important effect, while that of water–cement ratio and water-reducer agent is smaller, and HTFC admixtures have the smallest effect.

### 3.2. Analysis of Influencing Factors

**Figure 5:**
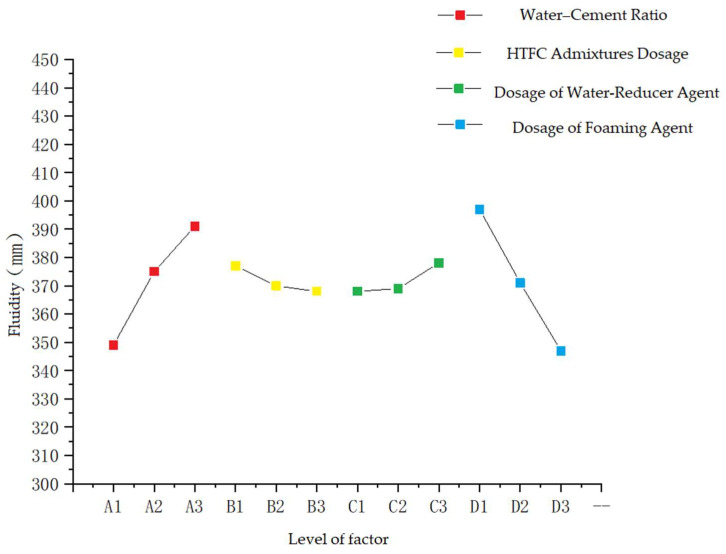
Relationship between factors and liquidity.

The “optimal conditions” in the process of orthogonal-test analysis are not absolute optimality, but for factors and levels. For intuitive purposes, the “relationship diagram between factors and fluidity” can be made by taking four factors, A, B, C and D, as abscissa and the fluidity of foamed concrete as ordinate, as shown in Figure 5.

Figure 5 shows that A factor (water–cement ratio) increases from 0.4 to 0.6, and the fluidity has a tendency to increase significantly in the process. The reason for which is that when the water–cement ratio is in this range, foamed concrete slurry can form a nearly circular blowhole with a relatively uniform aperture, and its resistance is small and appropriate so that the aperture also decreases gradually with the water–cement ratio bigger [27].

HTFC admixture has little influence on the fluidity of foamed concrete. With the increase in B factor, the fluidity decreases slowly.

The C factor can increase the fluidity of foamed concrete with the increase in the production of C factor under the condition of constant mix ratio. Because the polycarboxylate molecular structure of polycarboxylate superplasticizer accelerates the dispersion rate of cement in foamed concrete [28], the fluidity of foamed concrete slurry increases.

The microstructure and properties of foam are directly affected by the foaming mechanism of foaming agent. Mechanical foaming is a physical action. The foaming liquid produces bubbles under the action of mechanical stirring, and countless individual bubbles form foam. Bubbles use the special properties of surfactant or surfactant to wrap the air. The bubble wall is a stable and uniform double-electron-layer structure composed of surfactant or surfactant and water, and the bubble diameter is controlled at about 0.1 mm [29]. The opening and closing of bubble structure is directly related to the molecular microstructure and application method of surfactants and surfactants. Within the scope of the test mixture, with the increase in D factors (foaming agent), foamed-concrete fluidity shows a trend of decrease, because with the increase in foaming agent dosage, foaming rate becomes higher than that of slurry-condensation-sclerosis rate, and the mechanical strength of hole wall is low so that small and large holes are easily formed in the hardened cement paste, leading to poor porosity uniformity and fluidity [30]. Therefore, the fluidity can be better when the dosage is controlled within the range of 3–3.5%.

## 4. Engineering Application

### 4.1. Project Overview

The research results were applied to the foamed-concrete-filling project of the Yangtze River Shield Crossing Project of the China–Russia East-Route natural-gas-pipeline project. Three D1422 mm pipelines were laid in the Yangtze River Shield Tunnel, one for the China–Russia East-Route pipeline, one for Jiangsu coastal pipeline and one for the reserved pipeline shared by both parties. The project features long filling distance, large quantities and limited construction space.

**Figure 6:**
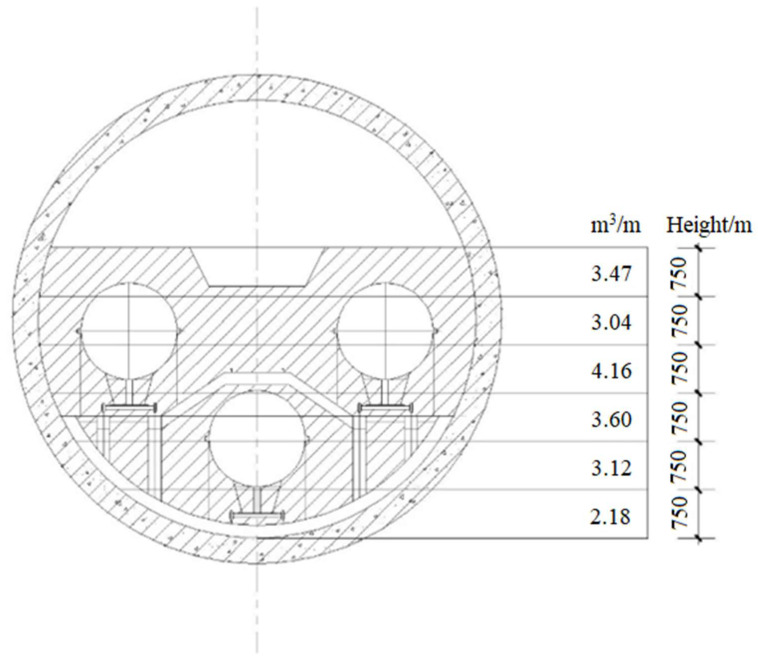
Schematic diagram of thickness of foamed concrete pouring layers.

The self-leveling-construction technology was adopted for site construction. Due to the long tunnel distance, large diameter, limited on-site preparation capacity of foam concrete in the confined space and the pouring affected by the fluidity of foamed concrete, the filling area of the tunnel was divided into pouring blocks and pouring layers to ensure the site-construction quality. The pouring layers are shown in Figure 6.

### 4.2. Construction Process Control Scheme

According to DB33T-996-2015 Technical Specification for Foamed Concrete Application on Highway, the requirement for fluidity of foamed concrete is 180 ± 20 mm. By referring to relevant engineering technical reports, the engineering requirement for the fluidity of foamed concrete is 350–450 mm. According to the influencing law of different factors on the fluidity of foamed concrete, the self-leveling-construction-process-control scheme of foamed concrete was proposed as follows: the range of water–cement ratio was controlled within 0.5~0.6, the admixtures content was controlled within 35~40%, the water-reducer-agent content was controlled at 1% and the foaming-agent content was controlled within 3~3.5%. According to the range-analysis results, the foamed-concrete-mix proportion was within this range, and the fluidity of foam concrete can reach 400 mm.

However, during construction, due to the long construction distance, some equipment could not reach the ideal construction position. The construction situation in the actual project was usually such that the internal structure of the tunnel was very complex and there were many irregular spaces, so the risk of manual construction was huge, and the existing construction technology and equipment could not achieve the best effect of self-leveling-foam-concrete construction. Therefore, this study referred to the existing technical methods and optimized the construction equipment on this basis so as to solve this dilemma and sent foam concrete to the construction site in a timely and accurate manner with high production.

### 4.3. Construction Equipment Optimization

In order to meet the requirement of foamed concrete self-leveling construction technology, this paper further designed a set of digital foamed-concrete-production-and-filling system suitable for long-distance and confined-space foam-concrete backfilling, and the scheme is as follows:

(1) Transportation trolley system setting: two sets of 20 t large-storage and transportation-tank transportation trolley were configured as the transportation trolley to transport bulk cement. The working mode was reciprocating and non-intermittent transportation. One set was transported after being filled, the other continued to load, and after being transported in place and unloaded, it returned to the bottom of the shaft to continue loading and then continued to transport so as to ensure that the bulk cement is transported to the production trolley timely and sufficiently;
(2) Production trolley system setting: the cast-in-situ foam concrete shall be prepared and used at any time, and the retention time shall not exceed 30 min. Therefore, a set of on-site production system of foam concrete was set on the electric track trolley, so that it can move freely in the long-distance tunnel. The system consists of a set of 20 t bulk cement storage tank, a set of 15 t bulk cement storage tank, a closed cement mixer, a cement foaming machine and a tractor. All equipment shall be installed horizontally to meet the needs of production and pouring construction in the confined space;
(3) Setting of pouring trolley system: the pouring trolley system was set at the front end of the foamed concrete production trolley, and the mixed foam concrete can be immediately pumped to the pouring trolley through the pipeline for pouring. The pumping wooden frame was set at the front end and both sides of the pouring trolley. The height of the wooden frame can be adjusted according to the pouring needs. The pumping pouring pipes were placed equidistant on the pumping wooden frame, and multiple pipes were poured at the same time. In combination with the digital control foam concrete production trolley system with the rear foam concrete conveying capacity up to 60 m^3^/h, the single-layer pouring in the pouring block can be completed in a shorter time to ensure that the foam concrete flows smoothly and evenly under the best flow state.

**Figure 7:**
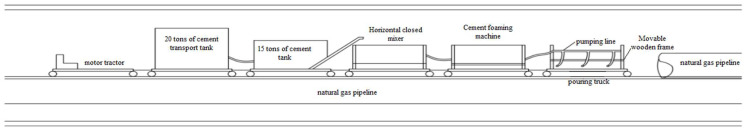
Schematic diagram of filling system for digital foamed-concrete production.

The complete set of digital automatic control system is composed of three parts: transportation, production and pouring trolley system. This system has the advantages of fewer errors and large output and is applicable to the featured requirements of the confined space and long-distance self-leveling construction of the project. The digital foamed concrete production backfilling system is shown in Figure 7.

## 5. Conclusions

Relying on the foamed-concrete-filling project of the Yangtze River Shield Crossing Project of the China–Russia East-Route natural-gas-pipeline project, the self-leveling method applicable to the construction of long-distance and confined-space tunnels filled with foamed concrete is studied. The influence of water–cement ratio, admixtures, additives and foaming-agent content on the fluidity of foamed concrete is analyzed. The results show that the factors affecting the fluidity [31] of foamed concrete are foaming-agent content > water–cement ratio > water-reducer agent > HTFC admixtures content. When the water–cement ratio was controlled within the range of 0.5~0.6, the HTFC admixtures content was 35~40%, the water-reducer agent content was 0.5~1% and foaming-agent content was 3~3.5%. Then the fluidity of foamed concrete reached 400 mm, meeting the requirements for foamed concrete fluidity in Technical Specification for Foamed Concrete Application on Highway [32] but far greater than the fluidity range in the specification. The reason is that it only meets the requirements in the specification, not the requirements of project construction. Therefore, improving the fluidity of foamed concrete is one of the core goals of this paper, which was realized by optimizing the mix proportion of foamed concrete [33]. High-fluidity foamed concrete does not need to be leveled manually in the construction process, and the high fluidity of foamed concrete makes it possible to fill the irregular voids in the confined space so as to reduce manual construction and achieve the effect of automatic leveling.

After a series of preparatory work, it is found that the foamed-concrete self-leveling-construction method has high requirements not only for the fluidity of foamed concrete but also for the on-site construction equipment. The existing construction technology and equipment could not achieve the desired effect. In order to meet the requirements of foam-concrete self-leveling construction technology, this paper further designed a set of digital foamed-concrete-production-and-filling system suitable for long-distance and confined-space foamed-concrete backfilling. The system adopted digital automatic control and had the advantages of fewer errors and large conveying capacity. The conveying capacity could reach 60 m^3^/h, which was suitable for the featured requirements of confined space and long-distance self-leveling filling of the project. Only when the mix proportion of foamed concrete was controlled to make the slurry meet the fluidity requirements and new construction technologies and equipment were used, could the foam concrete achieve better flow effect in the pouring process.

The research results were applied to the foamed concrete filling project of the Yangtze River Shield Crossing Project of the China–Russia East-Route natural-gas-pipeline project and achieved good application results in the project construction. On the basis of this project, this paper forms a theory about the foamed-concrete-self-leveling-construction method and construction equipment through systematic tests. This theory is not only applicable to the foamed-concrete-filling project of the Yangtze River Shield Crossing Project of the China–Russia East-Route natural-gas-pipeline project but also generally applicable to the foamed-concrete-filling project of the tunnel with long construction distance and complex internal structure. The novelty of this study lies in that, firstly, foamed concrete with a fluidity of 400 mm innovatively exceeds the maximum fluidity of traditional foam concrete, and a new maximum fluidity of foam concrete is obtained. Secondly, the existing construction equipment and technology can only be backfilled in short-distance tunnels. The layout of the equipment in the three construction processes of production, transportation and pouring is scattered and the operation is complex. Based on this, we innovatively design this equipment in a unified way to form a complete set of automatic foam-concrete production and backfilling system so that it can complete foam concrete backfilling in long-distance tunnels.

In a word, foam concrete appeared not late in China, but foam concrete is rarely used in tunnel filling in China. The digital foamed concrete production backfilling system designed in this study and the proposed mix proportion scheme of foam concrete are expected to provide a theoretical basis for the research of domestic advanced construction methods and technologies.

## Figures and Tables

**Table 1:** Physical property indexes of cement.

Number	Setting Time/min		Compressive Strength/MPa			Breaking Strength/MPa		
	Initial Set	Final Set	3d	7d	28d	3d	7d	28d
Ordinary Portland Cement	70	242	18.5	28.0	47.0	3.6	5.1	7.3

**Table 2:** Chemical composition of fly ash in HTFC admixtures.

Composition	SO_3_	MgO	CaO	Fe_2_O_3_	Al_2_O_3_	SiO_2_	Loss on Ignition
Content	0.66	1.09	3.53	4.04	30.37	50.76	3.32

**Table 3:** Technical indexes of polycarboxylate acid superplasticizer.

Appearance	Stacking Density/(g/L)	PH Value (20% Solution)	Sulfate Ion Content/%	Chlorine Ion Content/%
White	500~600	6~8	2.6	0.01

**Table 4:** Factors and levels in orthogonal test.

Group	Water–Cement Ratio (A)	HTFC Admixtures Dosage (B)	Dosage of Water-Reducer Agent (C)	Dosage of Foaming Agent (D)
1	0.4	35%	0.05%	3%
2	0.5	40%	0.5%	3.5%
3	0.6	45%	1.0%	4%

**Table 5:** Orthogonal-test table.

Group	Water–Cement Ratio (A)	HTFC Admixtures Dosage (%) (B)	Dosage of Water-Reducer Agent (%) (C)	Dosage of Foaming Agent (%) (D)
1	0.4	35	0.05	3.0
2	0.4	40	0.5	3.5
3	0.4	45	1.0	4.0
4	0.5	35	0.50	4.0
5	0.5	40	1.0	3.0
6	0.5	45	0.05	3.5
7	0.6	35	1.00	3.5
8	0.6	40	0.05	4.0
9	0.6	45	0.50	3.0

**Table 6:** Results of orthogonal-test data.

	A	B (%)	C (%)	D (%)	Fluidity (mm)
1	0.4	35	0.05	3.0	376
2	0.4	40	0.50	3.5	343
3	0.4	45	1.00	4.0	327
4	0.5	35	0.50	4.0	354
5	0.5	40	1.00	3.0	405
6	0.5	45	0.05	3.5	367
7	0.6	35	1.00	3.5	402
8	0.6	40	0.05	4.0	361
9	0.6	45	0.50	3.0	411

**Table 7:** Range analysis of orthogonal test.

Number		Factor			
		A	B	C	D
Level	K_1_	349	377	368	397
	K_2_	375	370	369	371
	K_3_	391	368	378	347
R		42	9	10	50
Optimal Level		A_3_	B_1_	C_3_	D_1_
Sequence		2	4	3	1

## Data Availability

The data used to support the findings of this study are available from the corresponding author upon request.
